# Positive Association between *TGFB1* Gene and Susceptibility to Idiopathic Scoliosis in Bulgarian Population

**DOI:** 10.1155/2018/6836092

**Published:** 2018-07-18

**Authors:** Svetla Nikolova, Milka Dikova, Dobrin Dikov, Assen Djerov, Alexey Savov, Ivo Kremensky, Alexandre Loukanov

**Affiliations:** ^1^Laboratory of Medical Genetics and Molecular Biology, University Hospital Lozenetz, Sofia University “St. Kliment Ohridski”, 1 Kozyak St., Sofia 1407, Bulgaria; ^2^University Orthopedic Hospital “Professor Boycho Boychev”, Medical University-Sofia, 56 Nikola Petkov Blvd., Sofia 1614, Bulgaria; ^3^National Genetic Laboratory, University Hospital “Maichin Dom”, Medical University-Sofia, 2 Zdrave St., Sofia 1431, Bulgaria; ^4^Molecular Medicine Center, Medical University-Sofia, 2 Zdrave St., Sofia 1431, Bulgaria; ^5^Graduate School for Science and Engineering, Saitama University, 255 Shimookubo, Sakura-ku, Saitama 338-8570, Japan

## Abstract

Idiopathic scoliosis (IS) is a common medical condition beginning in childhood and characterized by strong evidence for a genetic susceptibility to three-dimensional spinal deformity. The primary goal of the current case-control study is to examine the association between the *TGFB1* (-509C/T) functional polymorphic variant and genetic predisposition to IS in the Bulgarian population and the genotype-phenotype correlations in distinct case-control subgroups based on age at onset, family history, and gender. A total of 127 patients with primary scoliosis and 254 gender-matched control subjects were recruited. The mean Cobb angle was 53.8 ± 21.2°. Genotyping of cases and controls was performed using the TaqMan real-time amplification technique. The results were processed statistically using Pearson's Chi-squared test and Fisher's exact test with a value of *p* less than 0.05 as statistically significant. The polymorphic T allele and TT genotype were associated with a greater incidence of IS and can be considered as predisposing factors with a moderate effect on deformity development. The current results suggested that there was a genetic predisposition in early and late onset IS and familial, sporadic, and female cases. Nevertheless, replication studies are needed to reveal the relationship between the *TGFB1* locus and certain subtypes of IS in different populations.

## 1. Introduction

While mutations are the root cause of monogenic diseases and rare syndromes, functional genetic polymorphisms can often be associated with the etiopathogenesis of multifactorial diseases and common medical conditions. Association studies (candidate-gene and genome-wide case-control association studies) remain the leading approach for identification of genetic variants associated with complex human diseases. Idiopathic scoliosis (IS) is a common medical condition beginning in childhood and characterized by strong evidence for a genetic susceptibility to three-dimensional spinal deformity [1]. Adolescent idiopathic scoliosis (AIS) beginning after 10 years of age is the most common type of spine deformity [2].

*Transforming growth factor beta* 1 (TGFB1) is a multifunctional cytokine whose expression is associated with the physiological processes of growth, differentiation, regeneration, and stress response in many cell types [3–5]. Dickinson et al. [6] reported high transcript and/or protein levels in connective tissues during the periods of active morphogenesis, suggesting that TGFB1 is an important factor for their growth and differentiation. The results from two studies [7, 8] in AIS patients of Asian descent also indicated a marked increase in *TGFB1* expression level at the concave side of the spinal curve compared to the convex side. This phenomenon suggests the involvement of *TGFB1* as an etiological factor or a secondary factor in the development of the spine deformity. The increase of the TGFB1 level in the apical articular processes of the concave side of the curve in AIS may be due to the reconstruction of the extracellular matrix and the compensatory reactions which are caused by abnormal biomechanical forces, especially compressive stresses [7, 8]. In a study of 9 IS patients of European descent, no differences in *TGFB1* expression were found between paraspinal muscles of both sides of the curve [9].

A single nucleotide polymorphism (SNP), *TGFB1* (-509C/T; -1347C/T), affects transcription and plasma levels of the protein product. Shah et al. [10] found that the different expression levels are determined by the suppression of transcription through the activator protein AP1. *In vitro* and *in vivo*, the AP1 complex containing JunD and c-Fos was found to bind to the *TGFB1* promoter only in the presence of the wild type allele (-1347C). Thus, elevated levels of TGFB1 are associated with the polymorph allele (-1347T) due to a loss of negative regulation by AP1 [10].

A study in a Russian population sample (300 cases/300 controls) revealed a genetic association between *TGFB1* (-509C/T) and a predisposition to AIS. In the group of female patients, *TGFB1* (-509C/T) correlated with both the age of disease onset and curve severity [11]. A Chinese replication study (1251 cases/994 controls) did not confirm the association with the predisposition but with the mean Cobb angle in AIS patients [12]. The outcome measure of the aforementioned molecular genetic studies was the most common form of scoliosis—AIS. At the same time, there is limited available research on specific forms based on age at disease onset, family history, gender of the patients, and so on. Different genetic factors may be involved in the etiopathogenesis of early and late onset scoliosis as well as progressive and nonprogressive scoliosis, familial and sporadic cases, and male and female scoliosis. Wherefore, additional research is needed to clarify the possible contribution. The primary goal of the current case-control study is to examine

- the association between the *TGFB1* (-509C/T) functional polymorphic variant and genetic predisposition to IS in the Bulgarian population;
- the genotype-phenotype correlations in distinct case-control subgroups based on age at disease onset, family history, and gender.

## 2. Materials and Methods

### 2.1. Materials

A total of 127 patients with primary scoliosis and 254 gender-matched control subjects were recruited. For the aims of the current study, an informed consent was obtained from all individual participants or their parents in the case of minor patients. All work was conducted in accordance with the Declaration of Helsinki (1964). The responsible ethical committee of Medical University-Sofia approved the experiments (number 2987/2012).

The clinical diagnostic protocol included anamnesis, physical examination with Adam's forward bend test and a scoliometer, and radiographic studies of the spine in patients. The minimal value of the Cobb angle to define scoliosis was 10°. The mean Cobb angle was 53.8 ± 21.2°. Secondary scoliosis (congenital, neuromuscular, syndrome-related, etc.) was excluded. Thereafter, the cases were divided into three subgroups according to the disease onset: infantile idiopathic scoliosis (IIS)—up to 3 years of age (*n* = 4); juvenile idiopathic scoliosis (JIS)—from 4 to 10 years of age (*n* = 26); and adolescent idiopathic scoliosis (AIS)—from greater than 10 to 18 years of age (*n* = 97). The mean age of idiopathic scoliosis onset was 11.2 ± 2.9 years. Finally, the patients were divided into two subgroups by gender—females (*n* = 102) and males (*n* = 25) and according to family anamnesis—familial (*n* = 34) and sporadic (*n* = 93) cases.

The control group had the same gender distribution—females (*n* = 204) and males (*n* = 50). Anamnesis, physical examination, and previous available spinal roentgenographies excluded mild scoliosis among the control subjects. All the controls were selected among skeletally matured individuals over 18 years of age to exclude primary scoliosis at a later stage.

### 2.2. Methods

Automated magnetic bead-based extraction of genomic desoxyribonucleic acid (DNA) from peripheral venous blood samples by a chemagic DNA Blood 10 k Kit (PerkinElmer, Baesweiler, Germany) was performed using a chemagic Magnetic Separation Module I (PerkinElmer, Baesweiler, Germany) according to the manufacturer's instructions.

Real-time amplification by a TaqMan SNP Genotyping Assay available under assay ID C_8708473_10 (Thermo Fisher Scientific, USA) on an ABI Prism 7900HT Sequence Detection System (Thermo Fisher Scientific, USA) was used according to the manufacturer's recommendations for *TGFB1 (rs1800469)* genotyping. All samples were run in duplicate in 5 *μ*l of reaction mix containing 2 *μ*l of DNA (10 ng/*μ*l), 2.5 *μ*l of 2x TaqMan Genotyping Master Mix (Thermo Fisher Scientific, USA), 0.125 *μ*l of 40x TaqMan SNP Genotyping Assay (Thermo Fisher Scientific, USA), and 0.375 *μ*l of dH_2_O. TaqMan real-time polymerase chain reaction (PCR) was performed with an initial denaturation of 10 minutes at 95°C. The following thermal cycle was repeated 40 times: denaturation at 95°C for 15 seconds, annealing at 60°C for 60 seconds, and extension at 72°C for 15 seconds.

The results were processed statistically using Pearson's Chi-squared test for the general sample (*N* = 381) and the larger clinical subgroups (*N* > 300) or Fisher's exact test for the smaller clinical subgroups (*N* < 300) with a value of *p* less than 0.05 as statistically significant. Odds ratios (OR) and risk ratios (RR) with 95% confidence interval (CI) were also calculated (IBM SPSS 19.0, NY, USA).

## 3. Results

The genotype and allele frequencies of *TGFB1* (-509C/T) were analyzed in the general group and the distinct subgroups based on disease onset, family history, and gender. Each patient group was compared to the total number of controls except for both of the gender groups which were compared to twice as many gender-matched controls. We examined the possible genetic predisposition using the following inheritance models: codominant (TT versus CC), dominant (TT + CT versus CC), recessive (TT versus CT + CC), and allelic (T versus C). The genotypes were in Hardy-Weinberg equilibrium.

In the overall group, the frequencies of the homozygous TT genotype and polymorphic T allele of *TGFB1* (-509C/T) were higher in the cases than that in the controls (TT versus CT versus CC, *p* = 0.001, *χ*^2^ = 13.05 and T versus C, *p* = 0.0003, *χ*^2^ = 12.35, resp.). The homozygous TT genotype was associated with a 1.56 times higher risk (TT versus CT + CC, RR = 1.56, 95% CI: 1.22–2.00), and the T allele with a 1.23 times higher risk (T versus C, RR = 1.23, 95% CI: 1.11–1.38), for the development of idiopathic scoliosis in Bulgarian patients. The obtained results suggested that the TT genotype should be considered as a risk factor with a moderate individual impact (56% greater risk of deformity) as a part of the genetic predisposition to disease in the Bulgarian population.

In the subgroups of AIS, JIS, familial idiopathic scoliosis, sporadic idiopathic scoliosis, and females, the observed genotype and allele frequencies of *TGFB1* (-509C/T) differed also considerably between the cases and controls (*p* < 0.05, *χ*^2^-test or Fisher's exact test). The highest RR value was recorded in the subgroup of JIS (TT versus CT + CC, RR = 1.79, 95% CI: 1.23–2.6, Fisher's exact test). The four cases of IIS were excluded. By enrolling too few subjects, the study will not have enough statistical power to detect any difference (type II error). No statistically significant association was detected in the small subgroup of males (*p* > 0.05, Fisher's exact test).

The genotype and allele distributions are presented in Table 1. *P* values and ORs of the genotypes and alleles under the examined inheritance models are summarized in Table 2.

Then, we divided the female cases and significant association was detected in the subgroups with JIS, AIS and familial and sporadic forms of scoliosis (*p* < 0.05). *P* values and ORs of the genotypes and alleles under the examined inheritance models are summarized in Table 3.

**Figure 1:**
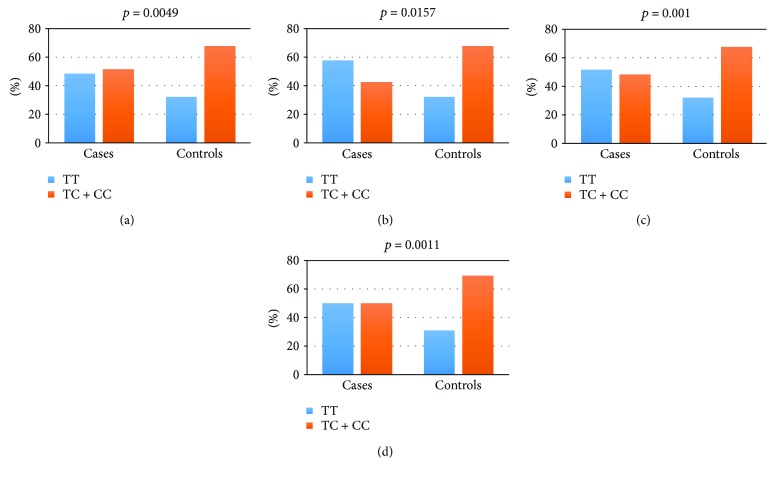
Recessive genetic model ТТ/(ТС + СС) in (a) adolescent idiopathic scoliosis (AIS), (b) juvenile idiopathic scoliosis (JIS), (c) nonfamilial idiopathic scoliosis (NFIS), and (d) females.

The obtained results reveal that the recessive model, having the lowest *p* value, best explains the inheritance pattern of the risk TT genotype. The recessive model in the four subgroups is presented graphically in Figure 1.

## 4. Discussion

The functional polymorphism *TGFB1* (-509C/T) was associated with AIS from two previous case-control studies in Asian and Caucasian population groups [11, 12]. In the current candidate-gene association study, the genotype and allele frequencies of *TGFB1* (-509C/T) were compared between patients and controls in a population of East European descent. In the general group, the polymorphic T allele and the homozygous TT genotype were associated with a greater incidence of idiopathic scoliosis and can be considered as a risk allele and a risk genotype, respectively, in the Bulgarian population (RR > 1.5).

The outcome measure of most of the association studies (candidate-gene or genome-wide association studies) was the most common form of scoliosis—AIS [13–20]. At the same time, available research on the early onset scoliosis (before 8–10 years of age) is still insufficient to establish genotype-phenotype correlations. Therefore, we included patients between 1 and 15 years of age at the onset of primary scoliosis. In the AIS subgroup, the results were similar to those in the total sample and in the JIS subgroup. A statistically significant association was also detected (Table 2). Larger replication studies are needed to confirm the novel association with early onset scoliosis predisposition.

Both in the familial and nonfamilial subgroups, the carriage of the T allele and TT genotype was associated with a susceptibility to idiopathic scoliosis (as shown in Table 2). No family history data from previous studies were available. The separation of cases according to this type of data was necessary because of the possible differences in genetic predisposition to familial and nonfamilial forms of a specific complex disease [21]. Our results suggested that there is genetic predisposition in familial as well as nonfamilial scoliosis, but the smaller sample of familial cases should be further expanded in order to support the conclusion of the current study.

Idiopathic scoliosis occurs more frequently in female than in male subjects [22–25]. Due to this reason, the case-control study on the association between *TGFB1* and AIS in the Chinese population included only female subjects [12]. In the current subgroup analysis stratified by gender, significant increasing susceptibility to deformity was detected in the general female group as well as in the female subgroups with JIS, AIS, and familial and sporadic forms of scoliosis (*p* < 0.05), but no significant association between *TGFB1* (-509C/T) and risk of deformity was found in males (see Table 2 and Table 3). Thus, the previously reported genetic association between *TGFB1* (-509C/T) and AIS predisposition in female cases from a population sample of European descent [11] was observed in Bulgarian patients.

Additional larger replication studies are necessary to confirm the relationship between the *TGFB1* locus and certain subtypes of IS in specific population groups and to detect a potential association between the *TGFB1* gene and idiopathic scoliosis in male patients. On the basis of the studies so far, genes encoding *TGFB* genes and their receptors (TGFBRs) may be included in the candidate-gene group involved in the etiology and pathogenesis of idiopathic scoliosis. Changes in the transcriptional profile of TGFBs and TGFBRs could influence the regulation of many signaling pathways potentially involved in the development and further progression of the deformity [26].

The potential clinical application of the findings for personalized treatment algorithms and improvement of the quality of care by allowing evidence-based management decisions motivated the development of genetic tests such as the ScoliScore AIS Prognostic Test in 2009. It was used to analyze the genotypes of 53 polymorphic markers associated with curve progression in Caucasian male and female patients with mild AIS (10–25° Cobb angle, 9 to 13 years of age) [27]. The test is no longer available after ScoliScore failed in 2 independent analyses conducted in Caucasian and Asian populations [28, 29]. In 2016, Bohl et al. demonstrated that the ScoliScore genetic test correlates with bracing outcome and may be appropriate for future bracing studies [30].

Additional genetic research could identify predisposing and modifying genetic factors of scoliosis and improve scoliosis prevention and treatment with early less invasive procedures. Providing new information could lead to more effective care with lower cost and fewer unnecessary radiographs and brace applications in the affected individuals [27].

## 5. Conclusions

On the basis of the present results, *TGFB1* (-509C/T) can be considered as a predisposing factor of idiopathic scoliosis with a moderate individual effect on deformity development in Bulgarian patients.

The current results may suggest that there is an association of the *TGFB1* (-509C/T) polymorphism with the susceptibility to IS in the female population with sporadic or familial IS and early or late onset IS.

Future extended association studies in different populations are necessary to confirm these findings and to examine the genetic correlates with the disease etiology in males.

The identification of prognostic molecular genetic markers is a contemporary approach that would permit an early treatment, including less invasive surgical or nonsurgical procedures.

## Figures and Tables

**Table 1:** Genotype and allele distribution in different subgroups of idiopathic scoliosis.

Group		Genotypes, *N*			Alleles, *N*	
		TT	TC	CC	T	C
General (*n*_1_ = 127, *n*_2_ = 254)	Cases	64	51	12	179	75
	Controls	82	126	46	290	218

AIS (*n*_1_ = 97, *n*_2_ = 254)	Cases	47	42	8	136	58
	Controls	82	126	46	290	218

JIS (*n*_1_ = 26, *n*_2_ = 254)	Cases	15	8	3	38	14
	Controls	82	126	46	290	218

FIS (*n*_1_ = 34, *n*_2_ = 254)	Cases	16	17	1	49	19
	Controls	82	126	46	290	218

NFIS (*n*_1_ = 93, *n*_2_ = 254)	Cases	48	34	11	130	56
	Controls	82	126	46	290	218

Males (*n*_1_ = 25, *n*_2_ = 50)	Cases	13	10	2	36	14
	Controls	20	22	8	62	38

Females (*n*_1_ = 102, *n*_2_ = 204)	Cases	51	40	11	142	62
	Controls	63	103	38	229	179

AIS—adolescent idiopathic scoliosis; JIS—juvenile idiopathic scoliosis; FIS—familial idiopathic scoliosis; NFIS—nonfamilial idiopathic scoliosis; *N*—number of genotypes or alleles; *n*_1_—number of cases; *n*_2_—number of controls.

**Table 2:** Inheritance models in different subgroups of idiopathic scoliosis.

Group	Genetic model	*P* value	OR (95% CI)
General	Codominant	0.0020	2.99 (1.46–6.11)
	Recessive	0.0006	2.13 (1.38–3.29)
	Dominant	0.0265	2.12 (1.08–4.16)
	Allelic	0.0003	1.79 (1.30–2.47)

AIS	Codominant	0.0036	3.30 (1.43–7.57)
	Recessive	0.0049	1.97 (1.22–3.18)
	Dominant	0.0219	2.46 (1.12–5.43)
	Allelic	0.0016	1.76 (1.24–2.51)

JIS	Codominant	0.1189	2.80 (0.77–10.2)
	Recessive	0.0157	2.86 (1.26–6.50)
	Dominant	0.4427	1.69 (0.49–5.89)
	Allelic	0.0258	2.04 (1.08–3.86)

FIS	Codominant	0.0122	8.98 (1.15–69.9)
	Recessive	0.1218	1.86 (0.90–3.84)
	Dominant	0.0241	7.3 (0.97–54.74)
	Allelic	0.0251	1.94 (1.11–3.39)

NFIS	Codominant	0.0170	2.45 (1.16–5.17)
	Recessive	0.0010	2.24 (1.38–3.63)
	Dominant	0.1615	1.65 (0.81–3.34)
	Allelic	0.0022	1.75 (1.22–2.50)

Males	Codominant	0.4508	2.6 (0.48–14.23)
	Recessive	0.4596	1.63 (0.62–4.27)
	Dominant	0.4804	2.19 (0.43–11.2)
	Allelic	0.2760	1.58 (0.75–3.29)

Females	Codominant	0.0072	2.80 (1.30–6.01)
	Recessive	0.0011	2.24 (1.37–3.65)
	Dominant	0.0778	1.89 (0.92–3.88)
	Allelic	0.0013	1.79 (1.25–2.56)

AIS—adolescent idiopathic scoliosis; JIS—juvenile idiopathic scoliosis; FIS—familial idiopathic scoliosis; NFIS—nonfamilial idiopathic scoliosis; *p*—probability value; OR—odds ratio; CI—confidence interval.

**Table 3:** Inheritance models in different female subgroups.

Females	Genetic model	*P* value	OR (95% CI)
General	Codominant	**0.0072**	2.80 (1.30–6.01)
	Recessive	**0.0011**	2.24 (1.37–3.65)
	Dominant	0.0778	1.89 (0.92–3.88)
	Allelic	**0.0013**	1.79 (1.25–2.56)

AIS	Codominant	**0.0094**	3.19 (1.29–7.86)
	Recessive	**0.0093**	2.02 (1.18–3.45)
	Dominant	0.0477	2.32 (0.99–5.45)
	Allelic	**0.0045**	1.76 (1.19–2.60)

JIS	Codominant	0.1332	3.62 (0.77–17.0)
	Recessive	**0.0085**	3.36 (1.31–8.62)
	Dominant	0.3892	2.06 (0.46–9.26)
	Allelic	**0.0211**	2.35 (1.12–4.92)

FIS	Codominant	**0.0179**	8.44 (1.07–66.8)
	Recessive	0.0859	1.96 (0.90–4.26)
	Dominant	0.0359	6.64 (0.88–50.3)
	Allelic	**0.0227**	1.98 (1.09–3.58)

NFIS	Codominant	0.0480	2.23 (1.00–5.00)
	Recessive	**0.0018**	2.37 (1.37–4.10)
	Dominant	0.3623	1.42 (0.67–3.02)
	Allelic	**0.0080**	1.72 (1.15–2.57)

AIS—adolescent idiopathic scoliosis; JIS—juvenile idiopathic scoliosis; FIS—familial idiopathic scoliosis; NFIS—nonfamilial idiopathic scoliosis; *p*—probability value; OR—odds ratio; CI—confidence interval.

## Data Availability

The data used to support the findings of this study are included within the article.
